# Copper Surfaces in Biofilm Control

**DOI:** 10.3390/nano10122491

**Published:** 2020-12-11

**Authors:** Inês B. Gomes, Manuel Simões, Lúcia C. Simões

**Affiliations:** 1 LEPABE, Department of Chemical Engineering, Faculty of Engineering, University of Porto, Rua Dr. Roberto Frias, s/n, 4200-465 Porto, Portugal; ibgomes@fe.up.pt; 2 CEB-Centre of Biological Engineering, University of Minho, Campus de Gualtar, 4710-057 Braga, Portugal; luciachaves@deb.uminho.pt

**Keywords:** antimicrobial, biofilms, cooling systems, copper materials, plumbing systems, health care units

## Abstract

Biofilms are structures comprising microorganisms associated to surfaces and enclosed by an extracellular polymeric matrix produced by the colonizer cells. These structures protect microorganisms from adverse environmental conditions. Biofilms are typically associated with several negative impacts for health and industries and no effective strategy for their complete control/eradication has been identified so far. The antimicrobial properties of copper are well recognized among the scientific community, which increased their interest for the use of these materials in different applications. In this review the use of different copper materials (copper, copper alloys, nanoparticles and copper-based coatings) in medical settings, industrial equipment and plumbing systems will be discussed considering their potential to prevent and control biofilm formation. Particular attention is given to the mode of action of copper materials. The putative impact of copper materials in the health and/or products quality is reviewed taking into account their main use and the possible effects on the spread of antimicrobial resistance.

## 1. Introduction

The presence of sessile microorganisms on abiotic surfaces can cause significant problems, representing considerable costs and negative effects in many different areas such as health and medical care units, water transport systems, ships and marine industry, heat exchangers and cooling systems. Sessile microorganisms may be divided in three main structures: adhered cells, biofilms and biofouling. Adhered cells correspond to the first stages of biofilm formation. After adhering on a surface, microbial cells start producing a matrix of extracellular polymeric substances (EPS) where they are embedded and protected from external stressful conditions (i.e., antimicrobials, nutrient starvation and hydrodynamics) and forming a structure called biofilm [1]. The process of biofilm formation may be described in several sequential steps: (1) the adsorption of organic and inorganic matter (presented in the bulk environment) on surface; (2) planktonic bacteria are transported to the surface and colonize it through physical, chemical and biological interactions. At this stage cells start the production of EPS and cell–cell signaling molecules (3), followed by the biofilm maturation and formation of a three-dimensional structure (4) [2]. On surfaces that are often cleaned and disinfected (surfaces and devices in hospitals and health care units) adhered cells are the role—EPS formation is negligible. Furthermore, biofouling is a critical and more complex phenomenon caused by the simultaneous accumulation of biological matter on surfaces (both micro- and macro-organisms) and the deposition of corrosion and crystallization products, suspended particles and other inorganic substances present in the neighboring environment [3]. In a recent review, Flemming [4] describes the huge impact of biofouling in a wide variety of systems: ion exchangers, membrane separation technologies, cooling systems, ship hulls, ship fuel systems, piping, sea chests, fuel and hydraulic systems, marine sensors, aquaculture, drinking water and plumbing systems, food, beverage and milk industries, paper industry, agriculture, cultural heritage, air condition systems and medical devices. The presence of sessile microorganisms in all these environments represents high economic impact for industries and systems, due to corrosion acceleration and coatings/materials deterioration (cooling systems [5], ships [6], drinking water and plumbing systems [7], cultural heritage [8], spacecraft equipment [9] and aircraft equipment [10]), increase of energy consumption (ships [11] and hydraulic systems [12]), deterioration/spoilage of products (food, beverage [13], dairy [14] industries, paper industry [15], water distribution systems [16] and agriculture [17]), yield/efficiency reduction (hydraulic systems [12] and membrane separation technologies [18]) and infection spread (cooling systems [5], air conditioning [19], aquaculture [20] and medical devices [21]). Further costs for biofilm/biofouling cleaning and disinfection should be considered [4].

The control of bacterial adhesion and consequent biofilm formation can be achieved by different strategies, depending on the environmental conditions and the application area. The most common strategies used to control biofilms are based on antimicrobials application, such as the use of disinfectants and biocides (on surfaces) or the use of antibiotics (on health-related biofilms). However, the use of chemical agents often fails and contributes to promoting bacterial resistance. Therefore, mechanical strategies can also be used for biofilm removal from different surfaces, i.e., the application of a mechanical load able to overcome the forces that keep biofilm intact on the surface, which will cause biofilm detachment [22]. Mechanical strategies are being applied in different areas such as on the control of dental biofilms (toothbrush or using fluid stress through high-velocity water jets, for example), cleaning procedures of medical devices (use of pressurized water brush) or in drinking water distribution systems (application of pipe flushing procedures). Additionally, the use of ultrasounds has also been used for biofilm control in food contact surfaces, mainly in combination with chemical strategies [23]. This strategy has also been used for biofilm control in chronical wounds, as described by LuTheryn, et al. [24]. The use of ultrasounds will promote the physical perturbation of biofilms, providing better conditions for antimicrobial action of chemical strategies [24]. Moreover, the use of surface materials with antimicrobial and antiadhesive properties is a very important strategy that has been extensively explored as a preventive strategy, being an alternative to conventional disinfection protocols or to antibiotics therapies. Balaure and Grumezescu [25,26] published an extensive two-parts review on the most recent developments in antimicrobial coatings for biofilm control. Antimicrobial surfaces may be classified as passive coating surfaces, used to prevent adhesion, which did not affect microbial activity but are responsible for the inhibition of microbial adhesion. These surfaces avoid bacterial adhesion since the material hydrophobicity, nanotopography, roughness and surface electric charge are carefully controlled to achieve this goal. Besides that, other modifications on surfaces that are associated with antimicrobial activity are defined by Balaure and Grumezescu [26] as active non-release-based antimicrobial coatings (i.e., silver, antibiotic/antiseptic and/or nitric oxide releasing systems) or as contact-killing surfaces (i.e., polycation-based coatings, antimicrobial peptides functionalized surfaces, nanosized metals and metal oxides, silica-based coatings, enzyme-based coatings and photoactivated-antimicrobial coatings). 

This review will focus on the use of copper-based surfaces, nanosized metals and metal oxides, as a potential strategy for biofilm prevention and control in different fields, such as in drinking water distribution systems, marine systems, food industry, medical devices, health care units and dental care. 

## 2. Antimicrobial Properties of Copper Materials

**Figure 1:**
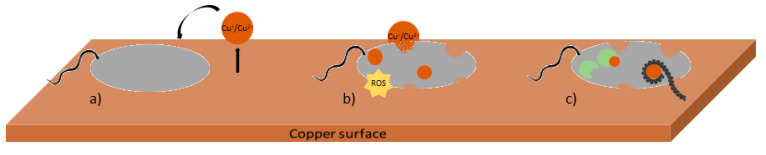
Sequential mechanisms involved in microbial-copper contact killing. (**a**) Release of copper ions (Cu^+^/Cu^2+^) from the surface and interaction with the bacterial envelope; (**b**) bacterial envelope damage caused by copper ions and by the formation of reactive oxygen species (ROS), leading to the presence of copper ions intracellularly and (**c**) damage of nucleic acids caused by copper ions and inhibition of enzyme activity.

The antimicrobial mode of action of copper surfaces has been extensively studied [27]. The mechanisms involved in contact killing may differ among microorganisms and on their state: planktonic, adhered on a surface or as multilayered biofilms [27]. The mechanisms related to the antimicrobial properties of copper surfaces are mainly due to the release of copper ions from metallic surfaces, but also may require the direct contact between microorganisms and surfaces [28]. Several mechanisms have been described as responsible for bacterial injuries and loss of activity when in contact with copper: cell envelope (outer and inner membrane) damage, oxidative damage caused by the production of reactive oxygen species (ROS), enzymatic inhibition and degradation of nucleic acids (Figure 1) [27,29]. However, the mode of action of copper and copper surfaces may alter according to environmental conditions particularly the presence of water, temperature, pH, presence of other metals or ions, oxidation state, surface roughness and the microorganisms present on the surface [27,30]. Therefore, several methods may be used to understand copper action in different conditions. Table 1 summarizes the main methodology used to evaluate membrane damage, intracellular copper, ROS formation, DNA damage and enzyme activity.

The antibiofouling properties of copper are commonly associated to the released copper ions that will cause cell damage due to alterations in protein/enzyme structure and activity [31]. Moreover, the copper interaction with biofilm is significantly different from that observed in planktonic cells. In biofilms, microorganisms are protected by a matrix of EPS. Therefore, the action of copper surfaces and copper ions will not only focus on microbial interaction but will also interfere with EPS. Nevertheless, the effect of copper on EPS formation is not well understood. Lin, et al. [32] demonstrated that copper ions in *Pseudomonas aeruginosa* biofilms were mainly located on the EPS matrix and only a small percentage was located intracellularly or in the cell membrane and/or wall. Some authors reported that the presence of copper might reduce the production of EPS by bacteria [33,34,35]. For example, Chari, et al. [33] extracted EPS formed by aquaculture pathogens after treatment with 10 ng/mL of copper nanoparticles and reported significant inhibition of EPS production by these pathogens. On its turn, Tabrez Khan, et al. [34] found that the production of EPS by oral bacterial population was inhibited after treatment with CuO nanoparticles and the inhibition of EPS production increased with increasing concentrations of CuO nanoparticles. Furthermore, Gomes, et al. [35] formed biofilms of *Stenotrophomonas maltophilia* on materials with different copper content and observed that biofilms formed on elemental metallic copper and on copper alloys with 57% and 96% of copper had lower amounts of extracellular proteins and polysaccharides than the biofilms formed on stainless steel. However, contradictory results of EPS production were also observed [36]. Miao, et al. [36] reported that the exposure to CuO nanoparticles increased the production of loosely bound EPS on wastewater biofilms. These EPS had higher content of proteins than polysaccharides and the CuO nanoparticles altered EPS structure and chemical composition, affecting significantly the functional groups of proteins and polysaccharides of loosely bound EPS [36].

## 3. Copper Surfaces on Biofilm Prevention and Control

**Figure 2:**
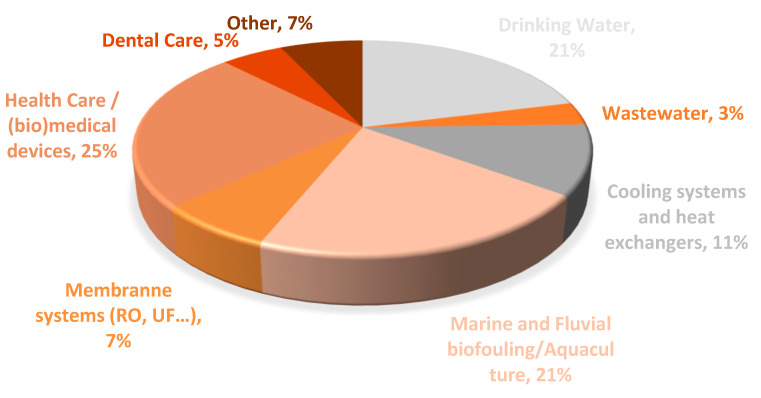
The main applications of copper surfaces according to the search obtained from SCOPUS database (29 September 2020) using the following keywords: “Copper” AND “Surfaces” AND “Biofilm” AND “Control”.

A review on the main applications of copper surfaces was performed using the Scopus database. A search for the words “Copper” AND “Surfaces” AND “Biofilm” AND “Control” on the titles, abstract and keywords of peer-reviewed manuscripts provided 152 results (last access at 29 September 2020). However, only 57 manuscripts (excluding reviews) described the use of copper materials for biofilm formation and control. That search demonstrated the wide range of copper surfaces applications (Figure 2). Most of these manuscripts described the use of copper materials in water systems (56%) such as in drinking water systems (21%), in marine and fluvial biofouling control (21%), cooling systems (11%) and wastewater (3%). The use of copper as antimicrobial and/or antibiofilm material in healthcare and (bio)medical devices is also an emerging issue, representing 25% of the original manuscript articles. Furthermore, the use of copper materials on membrane systems (i.e., reverse osmosis membranes) or in dental care procedures has also been explored by the scientific community, however, at a lower extent (5% and 7% of the manuscripts works). The remaining 7% of original studies were related with the petrochemical industry (1.7%) [45], construction sector–bricks (1.7%) [46], food contacting surfaces (1.7%) [47] and space flight materials (1.7%) [48].

A careful analysis of the recent publications proposes that the interest on copper nanostructured materials and copper nanoparticles to prevent and/or control biofilms in different areas is increasing.

In the following sections the application of copper materials on different areas was discussed, taking into consideration the results obtained from the search methodology described above. Other references of the authors’ knowledge not displayed in the search described were considered to complete this review.

## 4. Copper Surfaces in Marine Environment

Marine biofouling has a huge economic and environmental impact. Biofouling is responsible for an increase of roughness in ship hulls, increasing the drag force, which means higher consumption of fuel, resulting in higher associated cost and pollution. In fact, the biofouling will also imply cleaning procedures requiring time and economic investments. Nevertheless, the costs associated with the increase of fuel consumption are much higher than the cleaning-associated costs [49]. Several strategies have been developed to control biofouling in marine equipment [50]. Copper has been applied and studied as a possible strategy for marine biofouling prevention for several centuries [51]. More specifically, copper has been widely used in surface coatings or alloys composition for application in marine environments, namely in ship hulls, monitoring devices and also in aquaculture facilities. The use of copper alloys is an important strategy not only to improve the mechanical characteristics of metals, but it also may have significant impact in the antibiofouling activity. For instance, the presence of different alloy elements or impurities, such as zinc, lead, nickel, cobalt, zirconium or even molybdenum may play significant antimicrobial activity [52,53]. Aluminum can further improve the antimicrobial activity of dissolved copper [54]. For example Ford, et al. [55] demonstrated that after 25 days of metal immersion in a nutrient enriched pristine artic river, the microbial attachment was minimal on the studied copper alloy (90-10 copper-nickel). The use of copper on stainless steel (SS) alloys has been often studied for antibiofouling applications. For example, Kielemoes and Verstraete [56] investigated the bactericidal influence of copper-alloying of stainless steel (Cu-alloyed 3.72 wt % SS) on microbial colonization in natural waters. These authors demonstrated that copper in the SS matrix only impeded microbial adhesion for 48 h. Therefore, they proposed that Cu-alloyed SS could be applied for bactericidal purposes only in regularly cleaned surfaces. More recently, a novel copper-bearing 2205 duplex SS (2205-Cu-DSS) demonstrated interesting antimicrobial activity, reducing culturable *P. aeruginosa* in 33.1%, 56.0% and 70.3% after 1, 3 and 5 days of exposure in artificial seawater, respectively [57]. Furthermore, the analysis of *P. aeruginosa* biofilms using confocal laser scanning microscopy (CLSM) demonstrated that this alloy induced cell death and decreased biofilm thickness, supporting the potential use of this material for marine applications [57]. However, the combination of copper with SS results in a reduction of the alloy’s resistance to corrosion. Therefore, Li, et al. [58] evaluated the effect of copper addition on 2205 DSS on its resistance against pitting corrosion by *P. aeruginosa*. Analyzing 2205–3% Cu DSS by CLSM, it was found that this alloy had a strong resistance to pitting corrosion [58]. This was attributed to the copper-rich phases on the surface and to the release of copper ions, which will confer strong antibacterial properties to the alloy, inhibiting *P. aeruginosa* attachment [58]. Moreover, Liu, et al. [59] evaluated the corrosion resistance of 2205–3% Cu DSS against *Acetobacter aceti* biocorrosion. The results demonstrated effective biofilm inhibition and reduction in pitting depth in copper-2205 DSS in comparison to copper-free 2205 DSS [59]. Besides, high-entropy alloys (HEAs) have been developed in order to ensure antimicrobial properties and adequate mechanical characteristics. Zhou, et al. [60] designed a new HEA (Al_0.4_CoCrCuFeNi) containing copper—an antibacterial alloy with strong mechanical properties. These authors also suggested that the release of high concentration of copper ions from the HEA surface was the main responsible for biofilm prevention [60].

The use of antimicrobial/antibiofilm coatings or paints is the main strategy applied to control the development of biofilms in marine equipment. Despite the use of this kind of coatings/paints, biofilm development is not completely prevented/controlled and the toxicity of coatings/paints for non-target aquatic organisms has had special attention from the scientific community. Agostini, et al. [61] developed a long-term experiment in Patos Lagoon Estuary in Brazil in order to evaluate the effect of Zn- and Cu_2_O-based coatings on micro and macrofouling on steel surfaces and observed that Cu_2_O-based antibiofouling painted surfaces had the highest microfouling inhibition and the combination of Zn and Cu_2_O coatings resulted in the highest inhibition of invertebrates adhesion [61]. In a different study, copper oxide nanoparticles (NPs) with antibiofilm properties were investigated against *Staphylococcus lentus,* a copper tolerant marine bacterium [62]. These NPs were synthesized from copper nitrate by varying the concentrations of hexamine and cetyltrimethyl ammonium bromide (CTAB), and complete biofilm inhibition was observed with CuONPs at 1 mg/mL. However, capping the NPs with CTAB influenced NPs’ morphology and purity, but not their surface charge, reduced metal ion release and their antibacterial/antibiofilm properties. Uncapped NPs were more efficient in controlling biofilm formation than capped NPs [62]. Moreover, the antibiofilm properties of these NPs were due to a contact-killing interaction and copper ions release [62]. On its turn, the use of antibiofouling paints may induce antimicrobial resistance. Flach, et al. [63] investigated whether copper- and zinc-based antifouling paints can pose a risk for co-selection of antibiotic-resistant bacteria. The bacteria had increased resistance to heavy metals but also to tetracycline. That study also reported higher abundance of metal and biocides resistance genes and an enrichment of chromosomal RND efflux system genes, whereas mobile antibiotic resistance genes were not favored in the presence of the selected paints [63]. Therefore, it was proposed that heavy metal-based antibiofouling paints exert a strong selection pressure on marine microbial communities [63]. A study on the antimicrobial properties of biosynthesized copper particles (near400 nm) synthesized by *Shewenella indica* (isolated from a ship hull) showed that they were able to inhibit the growth of *Dessulfovibrio marinisedimis* [64]. Additionally, the influence of biofilm formation on monitoring equipment may hinder the required measurements. For example, diffusion gradient technique (DGT) in thin films is an important tool for monitoring reactive phosphorous in freshwater aquaculture effluents [65]. Biofilm formation on the surface of DGT devices interferes with phosphorous measurements. Pichette, et al. [65] suggested that the pretreatment of DGT membrane filters with copper would be useful to prevent biofilm formation in these devices as copper prevented algal colonization for 14 days post-deployment [65].

Moreover, it is important to highlight that the use of antifouling paints, such as those that contain copper, has been regulated by some European countries due to possible environmental consequences from copper leaching. In Sweden, Finland and Denmark the use of copper coatings in recreational vessels has been restricted [66]. In Finland and Denmark, the use of antifouling paints, including copper-containing paints, has also been restricted in freshwater bodies [67]. In the case of Finland, the rate for copper dissolution from recreational boat products cannot exceed 15 µg/cm^2^ per day [68].

## 5. Copper Surfaces in Plumbing Systems

The use of copper in drinking water (DW) transportation has been controversial. Some authors found important antimicrobial/antibiofilm properties from copper materials [37,69]. Other researchers demonstrated that the antibiofilm characteristics are not visible during long term experiments [70]. In addition to this discussion, copper corrosion and leaching to the transported DW is also a topic of concern. Metallic copper or copper alloys are commonly used in plumbing systems. However, the use of nanotechnology for the development of antibiofilm materials for plumbing systems has not been extensively explored.

The use of copper pipes demonstrated a significant effect on the reduction of biofilm formation [71,72], but it may also be responsible for alterations in the bacterial community in plumbing systems [73]. As an example, Silhan, et al. [74] assessed the effects of different materials in DW biofilm formation and found that 58-day old biofilms formed at 15 °C on copper pipes had lower density than biofilms formed on galvanized steel (GS), cross-linked polyethylene (PEX) and medium-density polyethylene (PE) pipes. Additionally, Silhan, et al. [74] demonstrated that the survival of *Escherichia coli* in biofilms developed on copper pipes was lower than on PE.

Other works focused on the use of copper to control specific waterborne pathogens, such as *Legionella pneumophila* [71]. Rogers, et al. [71] found that *L. pneumophilia* was absent in biofilms formed on copper pipes. Oppositely, Buse, et al. [75] found that *L. pneumophila* was more persistent in biofilms developed on copper surfaces, colonizing this material more effectively than PVC. A different study also demonstrated that copper is not effective in the inactivation of *L. penumophila* [76]. Gomes, et al. [37] used materials with different copper content (0%, 57%, 79%, 83%, 96% and 100% copper) to control *Acinetobacter calcoaceticus* and *Stenotrophomonas maltophilia* under growth conditions that simulated real plumbing systems and found significant reduction in bacterial culturability when copper materials were used. The use of an alloy containing 96% of copper presented promising results in the prevention of biofilm formation and regrowth [37]. In a different study, Gomes, et al. [35] evaluated the role of materials with different copper content (0%, 57%, 96% and 100% copper) in *S. maltophilia* biofilm formation and control by chlorination and/or mechanical stress. A rotating cylinder reactor was used to simulate the hydrodynamic conditions found in plumbing systems [35]. The results demonstrated that the use of materials with copper in its composition reduced the number of viable cells in biofilms in a similar or higher extent than the treatment with free chlorine at 10 mg/L [35]. It was suggested that copper alloys may play a positive impact in public health by decreasing the number of viable cells released into the transported DW during chlorine treatment. Moreover, the presence of copper materials was correlated with a decrease in the biofilm content of extracellular proteins and polysaccharides [35]. Oppositely, Wang, et al. [77] reported a 64-fold increase on extracellular polysaccharides in the EPS matrix when copper substrates were used in comparison to stainless steel, titanium or nickel. Moreover, an upregulation of metal transporter-related genes in bacteria attached to copper substrates was observed [77]. A recent study also proposed that the long-term bacterial survival in copper pipes was possible upon the induction of metal resistance mechanisms—an initial decrease on *Cupriavidus metallidurans* CH34 viability followed by a significant recovery after 48 h in contact with copper surfaces was described [78].

Most of the studies describing the effects of copper on biofilm control in plumbing systems are focused on the elemental copper and copper alloys. Nanotechnological uses of copper are starting to be explored. Sano, et al. [79] used a laboratorial biofilm reactor filled with tap water to evaluate the antibiofouling properties of silane coatings of dispersed silver and copper nanopowders. The copper silane coatings demonstrated antibiofouling effects as deposits of biological origin were only found on non-metallic silane coatings and were not observed on copper silane coatings. Apparently, the deposits observed on copper silane coatings had inorganic composition [79]. More recently, Baig, et al. [80] synthesized for the first time copper oxide-titanium dioxide nanocomposites using advanced pulsed laser ablation in the liquid (PLAL) technique for disinfection of waterborne biofilm forming bacteria. The synthesized nanocomposites demonstrated an enhanced antibiofouling and bactericidal activity against *P. aeruginosa* and methicillin-resistant *Staphylococcus aureus*. Copper oxide-titanium dioxide nanocomposites were not toxic for human cells (HEK-293) and were responsible for changes in the bacterial envelope. Therefore, PLAL synthesized copper oxide-titanium dioxide nanocomposites are of potential relevance for biofilm removal and/or pathogen inactivation in water distribution networks and/or in wastewater treatment plants [80]. It is obvious that the development of new materials based on copper may be a promising strategy to limit bacterial growth on surfaces used for water transport.

## 6. Copper Surfaces in Heat Exchangers and Cooling Systems

Biofouling development in cooling water systems such as heat exchangers, condensers and cooling towers represents a huge problem for industries (i.e. induce corrosion, reduce heat transfer efficiency and increase pressure drop) [4]. Moreover, the presence of biofilms in cooling towers may act as a reservoir of environmental pathogens that may constitute a severe public health risk. *L. pneumophila* outbreaks are from the most critical examples [81]. The advantages of using copper materials in cooling water systems has been investigated in order to understand the role of copper in the biofouling control. Copper and its alloys are widely used in industrial applications, namely in heating and cooling systems due to their interesting properties, such as high conductivity, corrosion resistance and mechanical workability, besides copper antimicrobial/antibiofilm characteristics. Li, et al. [82] studied the biocorrosion of mild steel (MS1010) and pure copper in the real and simulated cooling water environment. That study concluded that copper material was less susceptible to the corrosion in the presence of microorganisms than MS1010. Additionally, Schmidt, et al. [83] presented a comparative study to evaluate the ability of heat exchangers made of copper to prevent or control microbial growth. The study was conducted in a full-scale heating ventilation and air conditioning (HVAC) systems under normal flow rates and the results revealed that the concentration of bacteria and fungi in copper heat exchangers was lower than these detected in aluminum heat exchangers. Other studies presented innovative solutions for copper incorporation in cooling systems and heat exchangers, in order to reduce biofouling and the related problems [84,85,86]. For example, Vanithakumari, et al. [86] investigated biofilm resistance enhancement of cupronickel alloy (90-10 Cu-Ni) by modifying the alloy surface through sand blasting, pickling and coating with silane. *Pseudomonas* sp. was used as the model microorganism for adhesion studies providing information about the material resistance to biofouling. Silane based-coating on the sand blasted surface of cupronickel alloy was found to reduce bacterial adhesion in comparison to non-treated cupronickel alloy [86].

Regarding the application of nanotechnology for antimicrobial and antiadhesive surface improvement in heat exchangers/cooling systems, Vishwakarma, et al. [84] presented an alternative for titanium modification. Since biofouling/microfouling in cooling systems with titanium condensers is a significant problem, in that study the antibacterial properties of titanium surfaces modified with nanofilms of copper and nickel was evaluated. Two modifications were carried out by pulsed laser deposition, producing a copper-nickel bilayer and copper-nickel multilayers nano crystalline and thin films. The results demonstrated a better performance (decrease on bacterial attachment) of multilayer copper-nickel film than that obtained by bilayer deposition copper-nickel film. In a different work, copper-nickel deposition on titanium substrate was performed by three different techniques: DC magnetron sputtering, pulsed laser deposition and electroless plating [85]. The main goal of these modifications was the enhancement of antimicrobial properties of materials used in cooling systems. These modified surfaces were exposed to natural (seawater/water reservoir) and simulated conditions (laboratory pure cultures of microorganisms) to evaluate the antibiofilm properties and a reduction on biofilm formation was observed on the copper-nickel modified surfaces, regardless of the technique used for metal deposition [85].

## 7. Copper Surfaces in Membrane Systems

Membrane systems commonly used in desalination and wastewater treatment are highly affected by biofouling, compromising membrane performance. Biofouling causes declination on permeate flux, salt rejection and requires higher need of cleaning processes, increasing operating costs and reducing the membrane life time [87]. The use of copper coatings or nanoparticles in membrane systems has been explored in order to reduce and prevent biofouling [88]. Araújo, et al. [88] evaluated the impact of using polydopamine- and polydopamine-graft-poly (ethylene glycol)-coated feed spacers and membranes, copper coated feed spacers and commercially-available biostatic feed spacers on biofouling development in a membrane fouling simulator (MFS). The biofilm development on MFS was monitored through a pressure drop increase in the feed channel and by the assessment of biomass accumulation. The use of copper coated spacers retarded biofilm development but did not avoid its formation—reduced feed channel pressure drop and biomass accumulation was observed along the 15 days of experiment in MFS containing copper-coated spacers [88]. Copper-charged membranes were also investigated for microbial resistance and biofouling control [87]. Asapu, et al. [87] demonstrated that 93.2% of the uncharged membrane surface (control) was covered by biofilms, in contrast to copper-charged membranes whose covered surface area was about 67.9%. Furthermore, Guha, et al. [89] evaluated the use of polydopamine (PDA) membranes with catalytic metal oxide nanoparticles (copper oxide and manganese dioxide) anchored on its surface, revealing that CuO/PDA (at 8 or 80 ppm) coated membrane reduced *E. coli* adhesion and biofilm formation. Scanning laser confocal microscopy (CLSM) analysis demonstrated that specific biomass (µm^3^/µm^2^) was reduced by 88% and 95% in 8 and 80 ppm CuO/PDA membranes, respectively [89]. More recently, Wen, et al. [90] used nanotechnology to improve the characteristics of reverse osmosis membranes by developing a thin film nanocomposite membrane with the incorporation of a copper-based water stable metal-organic framework (CuBTTri) in the active layer. This new membrane had a reduced number of culturable *P. aeruginosa* on its surface in comparison to conventional membrane (pristine). These antibiofouling properties of the copper-based developed technology resulted in a significantly lower flux decline (30%) in comparison to the control membrane (70%). Such results were attributed to the depolarization of the bacterial membrane and to cell damage caused by the contact with the metal-organic framework—CuBTTri [90].

## 8. Copper Surfaces in Health Care Units

The presence of microbial cells in health-related surfaces may constitute a serious problem for patients’ health but also a significant economic burden. For example, only the medical-devices and surgical site bacterial infections caused by biofilms represent over a billion dollars per year to the healthcare system in the US [91]. Furthermore, in Europe, nosocomial infections may affect 3 million people every year, representing around 50,000 deaths/year [92]. Therefore, the development of innovative and effective strategies to avoid bacterial adhesion and biofilm formation in health-related surfaces (i.e., hospital and health-care units’ surfaces, medical devices and implants) represents a worldwide effort over the years. Copper is particularly relevant for the development of antibiofilm surfaces for health-care and biomedical applications, as minutely reviewed by Mitra, et al. [93]. Moreover, several studies tested metallic copper or its alloys for medical uses. For example, Furkert, et al. [94] investigated the antimicrobial activity of external fixation pins made out of stainless steel (SS), copper or titanium and SS coated with a polymer containing silver nanoparticles. These pins are normally used in bone fractures and deformations. The test pins were in contact with *Staphylococcus epidermidis* for 20 h and the uncoated copper pins demonstrated to be the most effective in biofilm prevention [94]. Other authors also studied the use of copper-containing alloys. Zhang and Liu [95] demonstrated that the Ti-10Cu sintered alloy has significant antibacterial activity against *E. coli* and *S. aureus.* These authors further treated the alloy by sand blasting (SB), sandblasted and large-grits acid etching (SLA) and alkaline treatments (AH). SB and SLA produced a rough surface covered by TiO_2_, with lower corrosion resistance and increased release of titanium and copper ions. On its turn, AH treatment formed a smooth and microporous surface with the TiO_2_/titanate layer more resistant to corrosion but also with increased release of titanium and copper ions [95]. Nevertheless, the treatments (SB, SLA and AH) did not affect the antibacterial properties of Ti-10Cu alloys against *S. aureus*, proposing that bacterial inactivation may be related to ion release [95]. Sun, et al. [96] also presented the austenitic 317L-Cu stainless steel (317L-Cu-SS) alloy as potential material for medical use as it inhibited sessile *S. aureus* by 99% [96].

The use of copper coatings or impregnation in other materials has also been explored as alternative antiadhesive or antimicrobial surfaces. The development of these kinds of materials aims to prevent biofilm formation and related infections, using more economical materials than elemental copper. Copper and copper-silver coatings were used by McLean, et al. [97] to reduce bacterial activity in different catheter materials. Mauerer, et al. [98] and Norambuena, et al. [99] coated titanium alloy Ti6A14V with 4-fold copper-titanium oxide (CuTiO_2_) and titanium-copper oxide (TiCuO), respectively, and evaluated their antibiofilm activity. Ti6A14V disc coated with a thin film of TiCuO with 80% copper content reduced *S. epidermidis* biofilm by 2.5 log of colony forming units (CFU). This material also demonstrated lower toxicity in normal human osteoblast [99]. Mauerer, et al. [98] further investigated the antibacterial effect of the Ti6A14V spacer coated with 4×CuTiO_2_ in an animal model, in order to simulate acute periprosthetic infection by *S. aureus.* The materials were implanted into the femoral condyle of rabbits and two weeks after the implementation of copper-titanium oxide coated spacers a decrease on the rabbits’ infection rate from 90% to 41.7% was observed [98]. Zang, et al. [91] used a copper-based (CuBTTri) metal-organic framework dispersed in a polymer solution to coat medical circulation tubing and to control bacterial adhesion and found reductions in *S. aureus* adhesion higher than 50% [91].

The impregnation/incorporation of copper on different polymers has also been used in different works. Wood, et al. [100] incorporated copper and cobalt within a polymeric matrix (unhardened Trylon resin) and found enhanced activity of oxidizing biocides (hydrogen peroxide and potassium monopersulfate) against *P. aeruginosa* biofilms. For example, the concentration of biocides needed to inactivate biofilms by 90% was reduced from 2.2 and 1.1 (biofilms formed on polymer surfaces without incorporation of metals) to 0.375 and 0.003 mg/L (biofilms formed on the presence of polymers with copper incorporated) of hydrogen peroxide and potassium monopersulfate, respectively. Therefore, Wood, et al. [100] presented copper and cobalt as catalysts of oxidizing biocides, reducing the concentration of biocides needed to effectively control *P. aeruginosa* biofilms. Boutin, et al. [101] demonstrated that the combination of algal lipidic extracts (from *Spirulina platensis*) with copper-alginate nanocarriers potentiated the antibiofilm activity of the algal extract against *Candida* species biofilms. Many other authors used copper in nanotechnological applications to improve antibacterial and antibiofilm properties of surfaces. Parrott, et al. [102] created films with various densities of copper NPs and evaluated their antimicrobial activity against bacteria commonly associated with nosocomial infections with promising results in the control of *Streptococcus pyogenes* and *S. aureus* biofilms. Copper particles were incorporated into nanofibers with the purpose to control wound biofilms, when incorporated in wound dressings [103]. Nanofibers containing copper particles reduced *P. aeruginosa* and *S. aureus* biofilms by 41% and 50%, respectively. These results reinforce the possibility of using copper-containing nanofibers in wound dressing to prevent biofilm development [103]. Moreover, Borkow, et al. [104] developed non-stick dressings composed of a highly absorbent internal mesh fabric and an external non-woven fabric impregnated with 2.65% (w/w) copper oxide particles. These authors reported promising broad-spectrum activity against bacteria and fungi and no adverse reactions were not observed in rabbit and porcine models. Furthermore, the copper-containing wound dressings demonstrated long term antimicrobial activity. More recently, Singh, et al. [105] also used a poly-acrilic acid (PAA) coated with 66–150 nm copper NPs and found that *P. aeruginosa* was highly resistant to copper ions and copper NPs. However, the analysis through scanning electronic microscopy (SEM) demonstrated that cell morphology changed in the presence of copper NPs. These NPs also downregulated genes involved in the development of the biofilm matrix, in bacterial motility, efflux mechanisms, the synthesis of membrane lipoprotein and DNA replication. In contrast, both copper NPs and copper ions upregulated copper resistance and biofilm dispersion genes. Despite that, copper NPs were highlighted as an important strategy to prevent nosocomial infections [105].

Nanotechnology has also been used in paint formulation. Tripathy, et al. [106] used CuO quantum dots synthesized by low temperature solution process, on the formulation of paint, building a quanta-CuO thin film on glass samples. This paint demonstrated high contact-killing capacity against *E. coli* and *S. aureus* biofilms being proposed as a promising coating for biomedical purposes. The high antibacterial/antibiofilm activity was related to the generation of intracellular reactive oxygen species (ROS), involved in bacterial cell death. LewisOscar, et al. [107] further suggested the use of copper NPs as coating agents on surgical devices and medical implants, since they found that copper NPs at 100 ng/mL (under bactericidal concentration) reduced *P. aeruginosa* biofilms by 94%. Additionally, LewisOscar, et al. [107] also demonstrated a decrease on surface hydrophobicity and on the production of extracellular polysaccharides, which highly contribute to a reduction on the ability to form biofilms.

Currently, the scientific community is also focused on the development of ecofriendly processes to synthesize NPs for biomedical approaches [31]. Punniyakotti, et al. [31] used plant (*Cardiospermum halicacabum*) extracts to reduce copper ions on the copper NPs production, demonstrating their antibiofilm activity. Copper NPs at 0.1 mg/mL reduced *E. coli, S. aureus* and *P. aeruginosa* biofilms in 78%, 72% and 79%, respectively [31]. Lotha, et al. [108] synthesized biogenic copper NPs with purified isoquercitin and cassinopin from *Crotalaria candicans* and demonstrated that those NPs were effective on the control of multidrug resistant *S. aureus*. Biogenic copper NPs altered bacterial membrane permeability and reduced surface hydrophobicity [108]. Biogenic copper-silver/silver-copper nanocomposites were also presented as antimicrobial and antibiofilm materials against *E. coli, P. aeruginosa* and *S. aureus* [109]. Singh, et al. [110] developed an antibacterial surface using copper nano-whiskers deposited by molecular beam epitax. The antibacterial effect was attributed to the hydrophobic pinning of water droplets in the Wenzel regime, which caused cell injury and consequently cell death. Copper nano-whiskers significantly inhibited *E. coli* biofilms [110].

The resins used in dental restoration may favor biofilm development and consequently increase the occurrence of secondary caries [111]. Therefore, copper has been incorporated in resins used for dental restoration. Zajdowicz, et al. [111] synthesized a novel copper-catalyzed azide-alkyne cycloaddition (CuAAC) based resins and composites and found that CuACC reduced biofilm formation, protecting dental restorations for longer periods in comparison to other commonly used resins [111].

The use of copper in high-touch surfaces in hospital equipment (such as beds and door handles) is known to decrease the number of viable bacteria on surfaces, reducing the risks from the spread of hospital-acquired infections [112,113]. Schmidt, et al. [112] monitored five high-touch intensive care bed surfaces by routine culture in order to assess the effect of copper surfaces (U.S Environmental Protection Agency registered as antimicrobial copper) on microbial burden. The authors demonstrated that despite daily cleaning and disinfection, the bacterial counts in plastic beds’ surfaces (used as the control) exceeded recommended values (2.5 log CFU/cm^2^). However, the use of copper materials in bed high-touch surfaces reduced significantly the bacterial counts [112]. Burke and Butler [114] also demonstrated that the use of copper-impregnated composite hard surfaces, bed linens and patient gowns on healthcare units reduced the incidence of nosocomial infections, the occurrence of *Clostridium difficile* infections and also the occurrence of infections caused by multidrug resistance microorganisms. Furthermore, Chatterjee, et al. [115] examined the effect from the impregnation of 16–20% copper oxide in a polymer-based resin on the bacterial contamination of high-touch surfaces (bedrails, footrails, tray tables and sinks) in patient rooms in an acute care hospital. In that study, samples were taken 3 times per day during a 3-day period in 32 rooms, 16 of them containing the copper-impregnated materials. The obtained results demonstrated that the use of copper on high-touch surfaces reduced microbial burden [115]. Moreover, a different study conducted by Coppin, et al. [116] showed persistently lower microbial burden on a copper-impregnated tray table in occupied patient rooms after thorough initial disinfection over a 30-h sampling period, in comparison to standard non-copper surface. Monk, et al. [117] impregnated Cupron CuO on non-porous surfaces of hospital settings in order to reduce the incidence of nosocomial infections. For that, the authors tested countertops composed of homogenous blends of polyester, acrylic alloys and fillers, inert pigment and dyes, with or without Cupron’s 16% CuO (w/w). These countertops were able to kill 99.9% of some hospital pathogens such as *S. aureus*, *Enterobacter aerogenes*, *P. aeruginosa*, methicillin resistant *S. aureus* and *E. coli* 0157:H7 [117]. On its turn Colin, et al. [118] evaluated the effect of copper alloys on microbial burden in five long term care facilities in France. For that, half of the original doors’ handles were replaced by 90% copper alloys and half of the original handrails were replaced by 70% copper alloys. The authors demonstrated that copper alloys were promising materials to avoid the spreading of environmental bacterial contaminations in health care facilities [118]. Moreover Salgado, et al. [113] demonstrated that the use of copper alloys in intensive care units’ rooms reduced the rate of hospital-acquired infections and the rate of surface colonization with methicillin-resistant *S. aureus* and vancomycin-resistant *Enterococcus*. The use of copper alloys may influence the material properties and special attention should be given to alloy elements in order to improve the desired characteristics of copper and while avoiding further negative impacts. For example, brasses (copper and zinc) can be used in hospital door furniture due to their proved antimicrobial activity, which can be tuned by changing the copper-zinc content. However, although the copper-nickel combination has important antimicrobial activity and provides strong mechanical properties of the material, nickel is a known allergen and is not recommended for high-touch surfaces [119].

The use of copper-impregnated textiles in hospital facilities has no direct impact in biofilm formation. However, it is important to highlight their potential to prevent hospital-acquired infections. Marcus, et al. [120] demonstrated that copper oxide-impregnated textiles reduced significantly the nosocomial-infection indicators (i.e., the use of antibiotics, period for treatment and fever days).

## 9. Other Applications for Copper Surfaces

Copper is a widely used material due to its interesting workability characteristics and corrosion resistance. In addition to the applications described above, the antibacterial and antibiofilm properties of copper are relevant for many other applications such as in the food industry [121], fuel and oil facilities [45], construction sector [46] or even in space flight or space station equipment [48]. Dygico, et al. [122] tested different materials that can be found in the mushroom production environment such as SS, aluminum, rubber, polypropylene, polycarbonate, concrete, borosilicate glass and copper and found that the use of copper reduced significantly the number of culturable *Listeria monocytogens* adhered on the surface [122]. Copper-based nanotechnology was tested for the development of safe food-contact surfaces [47,121]. Ghasemian, et al. [121] coated glass and SS surfaces (commonly found in food industry) with copper NPs and found a significant reduction in bacterial adhesion on coated surfaces. More recently, Wang, et al. [47] developed a nano CuO film on copper foil for in situ generation of reactive chlorine species (RCS) for biofilm eradication from food-contact surfaces.

Biofilm development in buildings is a significant concern as bacteria, fungi and algae in building biofilms are responsible for biodeterioration and may be related to some health issues such as allergies caused by fungal pathogens [123]. Gámez-Espinosa, et al. [46] developed antifungal additives with copper- and silver-NPs synthesized with an aqueous extract of *Senra occidentalis* L. A functionalized sol–gel-based product was produced to apply on bricks in order to avoid biodeterioration.

Nanomaterials have been introduced in the fuel/oil industry to control microbial growth and consequently prevent microbiologically influenced corrosion. Kalajahi, et al. [45] used copper nanoparticles doped carbon quantum dots nanohybrid (Cu/CQDs) as a (bio)corrosion inhibitor against *Desulfovibrio* sp.

Biofilm development has also been reported in space stations such as Soviet/Russian (Salyust and Mir), American (Skylab) and International (ISS) space stations. In this field, biofilms may threaten materials and key equipment like space suits, water recycling units, radiators or even navigation windows. Furthermore, biofilm formation may also increase the risk for crew infection and illness. All these problems reinforce the need for biofilm studies and for the development of control strategies to enhance microbiological safety, allowing long-duration human space missions [124]. Hahn C. [48] evaluated the possibility from using oxidized copper layers and copper surfaces in space flight in order to avoid biofilm development. A higher antimicrobial activity against *E. coli* and *S. aureus* was observed for the cuprous oxide layer than for pure copper surfaces. Such result proposes that copper-containing surfaces increase the production of ROS and promotes high bacterial inactivation [48].

## 10. Limitations from Copper Use

Although copper has been widely used as an antimicrobial surface for the most varied applications, a number of limitations have been reported. Several studies focused on the role of copper on antimicrobial resistance acquisition and spread [78,125,126]. The resistance to copper and the associated coselection for resistance to other antimicrobials may affect copper surfaces efficiency and safety. These resistance mechanisms are based on horizontal gene transfer [125] and several studies demonstrated that copper resistance may also coselect for antibiotic resistance. Silveira, et al. [127] demonstrated for the first time the cotransfer of ampicillin resistance along with copper tolerance genes among *Enterococcus* spp. from different origins (animals, humans, food and environmental samples). Furthermore, horizontal gene transfer of copper resistance along with other antibiotic resistance genes has been observed in other microorganism (such as *S. aureus, Salmonella typhimurium* and *Klebsiella pneumoniae*) obtained from different sources (soils, aquaculture, wastewater and drinking water) [126].

The use of copper surfaces in aquatic environments may be limited by pitting corrosion and copper leaching [37,128,129,130]. Copper corrosion products and copper leaching have significant impact in water quality [130]. Furthermore, the presence of copper corrosion products may persist in the biofilm matrix [131] and promote the formation of disinfection byproducts in drinking water networks when biofilms are present [128]. Additionally, copper ions from agricultural practices and other sources such as aquaculture and marine antifouling treatments or industrial effluents and copper NPs used in a wide variety of products may accumulate in the environment (soil, water and sediments). Therefore, copper ions and NPs may become a potential threat to the environment. Several studies evaluated the impact of copper NPs on microbial communities in sediment biofilms and in wastewaters [36,132,133,134,135]. The presence of copper NPs in aquatic sources may cause negative effects in biogeochemical processes [135]. Their presence may also shape the biofilms formed by wastewater bacterial communities, influencing the structure and the predominance of particular bacterial genera [134]. Recently, Yang and Wang [132] demonstrated that copper NPs and copper ions may significantly impact the aquatic biota, being able to bioaccumulate and cause toxic effects in barnacle larvae.

NPs can easily penetrate the membrane of mammalian cells [136]. The topical use of CuO NP induced inflammatory cytokine secretion and necrosis in human skin organ cultures [137]. The use of copper NPs in dentistry has been widely explored. However, the information about possible toxic effects is not completely understood. Short-term studies did not identify significant toxic effects caused by metal NPs in dentistry, nevertheless, the material will remain in a patient mouth for long periods [138]. Furthermore, copper toxicity is known for particular organs, as reviewed by Agnihotri, et al. [138] (i.e., neuromuscular toxicity, DNA damage and cellular apoptosis in lungs, increased oxidative stress in lungs and kidneys), increasing the concerns from the long-term effects of copper NPs on human health. The use of Cu NPs in the food industries or food products should also be adequately analyzed in order to fill the gap of knowledge in this field, and to define threshold limits for food quality and safety [136,139].

## 11. Conclusions

Undesired biofilm formation represents a significant economic burden regardless of the industry or economic area where they are developed. Therefore, innovative and effective strategies for biofilm prevention and control are needed. Copper materials have been investigated as potential innovative surfaces with antimicrobial activity for different applications, mainly in water systems such as in drinking water systems, in marine and fluvial biofouling control, cooling systems and wastewater. Furthermore, copper materials have also been applied in medical devices and high-touching surfaces in health care institutions reducing the spread of nosocomial infections.

Since conventional metallic copper has demonstrated some limitations for different applications, such as high costs, copper leaching and/or pitting corrosion, the scientific community has been exploring innovative alternatives and treatments to improve the results from copper applications. For instance, in marine applications, plumbing systems and heat exchanger, research has been focused mainly on copper alloys modification in terms of composition but also in terms of metallic treatments such as sand blasting, pickling and coatings. On its turn, in health care units and biomedical application and in the design for filtration membranes, besides the investigation on metallic alloys, nanotechnology has been extensively used for surface modification. The impact of copper materials on the prevention of bacterial adhesion and on the retardation of biofilm formation is undeniable, despite some controversial results for long term applications. However, a modest amount of information is available about the effects of copper materials on EPS formation in biofilms. Despite promising results on the control of bacterial adhesion and biofilm formation by using innovative copper materials, there are some limitations that should be considered when new strategies for copper application are developed, such as copper leaching and possible ecotoxic effects and the development and spread of microbial resistance to copper and the cross-resistance to antimicrobial products.

## Figures and Tables

**Table 1:** Examples of methodology applied to assess the mode of interaction of copper with microbial cells.

Mechanism of Action	Methodologies Used
**Membrane damage**	Fluorescence probes—microscopy or fluorometry: Live/Dead staining—SYTO9 (green) is able to penetrate the intact cell membrane and propidium iodide stains in red cells with a damaged envelope [37,38] Labeling intracellular copper ions (CS1 sensor) [29] Inductively coupled plasma measurement of intracellular copper [39] Membrane depolarization—using fluorescent probes as rhodamine and BacLight Bacterial Membrane Potential Kit containing oxa-(DiO) carbocyanine dye [40] Respiration activity—CTC (5-cyano-2,3-ditolyl tetrazolium chloride) redox dye [41]
**ROS formation**	Nitroblue tetrazolium (NBT) assay (absorbance measurement at 620 nm) [42] Dichloro-dihydro-fluorescein diacetate, H2DCFDA (excitation wavelength: 485 nm and emission: 530 nm) [37] Evaluation of copper antimicrobial action in the presence and absence of ROS quenchers
**DNA damage**	Comet assay—single cell gel electrophoresis [38] DNA integrity assessment by SYTO9 nucleic acid stain [41,43] Quantitative polymerase chain reaction (qPCR) [39] DNA fragmentation assay with SYBR gold staining [41,44] Mutagenesis assay—thyA [39] and CycA [29] mutants
**Enzymes activity**	Catalase, hydroperoxidase and β-galactosidase assays [39]
